# Hyperplastic Growth of Pulmonary Artery Smooth Muscle Cells from Subjects with Pulmonary Arterial Hypertension Is Activated through JNK and p38 MAPK

**DOI:** 10.1371/journal.pone.0123662

**Published:** 2015-04-23

**Authors:** Jamie L. Wilson, Jun Yu, Linda Taylor, Peter Polgar

**Affiliations:** Department of Biochemistry, Boston University School of Medicine, Boston, Massachusetts, United States of America; Nippon Medical School Graduate School of Medicine, JAPAN

## Abstract

Smooth muscle in the pulmonary artery of PAH subjects, both idiopathic and hereditary, is characterized by hyperplasia. Smooth muscle cells (HPASMC) isolated from subjects with or without PAH retain their in vivo phenotype as illustrated by their expression of alpha-smooth muscle actin and expression of H-caldesmon. Both non PAH and PAH HPASMC display a lengthy, approximately 94h, cell cycle. The HPASMC from both idiopathic and hereditary PAH display an abnormal proliferation characterized by continued growth under non-proliferative, non-growth stimulated conditions. This effector independent proliferation is JNK and p38 MAP kinase dependent. Blocking the activation of either abrogates the HPASMC growth. HPASMC from non PAH donors under quiescent conditions display negligible proliferation but divide upon exposure to growth factors such as PDGF-BB or FGF2 but not EGF. This growth does not involve the MAP kinases. Instead it routes via the tyrosine kinase receptor through mTOR and then 6SK. In the PAH cells PDGF-BB and FGF2 augment the dysregulated cell proliferation, also through mTOR/6SK. Additionally, blocking the activation of mTOR also modulates the MAP kinase promoted dysregulated growth. These results highlight key alterations in the growth of HPASMC from subjects with PAH which contribute to the etiology of the disease and can clearly be targeted at various regulatory points for future therapies.

## Introduction

Pulmonary arterial hypertension (PAH) is a devastating disease of the pulmonary vasculature which is ultimately fatal and presently with limited treatment. A principal pathogenic event of the disease is the thickening of the smooth muscle media and invasive proliferation of smooth muscle cells (SMC) into the intima and into multiplex regions of the blood vessel [1]. This proliferation leads to hypertrophy of the vasculature and contributes to sustained elevation in pulmonary vascular resistance and increased pulmonary arterial pressure [2]. Presently this hypertrophy has not been brought under control therapeutically.

To address this dilemma smooth muscle cells (SMC) from pulmonary arteries (PA) of patients with PAH in primary cultures have provided a number of insights into their proliferative mechanisms in vivo. Studies on human pulmonary artery smooth muscle cells (HPASMC) from PAH patients have described increased PAH HPASMC growth in response to stimuli such as TGFβ, BMPs [3] and serotonin [4]. These stimuli were shown to enact their growth responses through MAP kinases [5–7]. Other studies have implicated physiologic factors, such as increased intracellular Ca^2+^ [8, 9], secretion of pro-inflammatory cytokines [10], miRNA dysregulation [11], dysregulated serotonin transport and expression [12, 13] and altered growth factor expression [14, 15] as promoting proliferation in PAH HPASMC. More recently, tyrosine kinase receptors, such as PDGFR, EGFR, and FGF2R have been proposed responsible for the increased HPASMC growth in PAH [14–17]. In fact, clinical trials evaluating the efficacy of PDGFR signal inhibitor, imatinib, on PAH have been carried out [18, 19]. Imatinib is a modulator of phosphorylation sites of ABL and the PDGF receptor [20]. However, treatment of PAH with imatinib has had only limited success suggesting that the growth factor has only a limited role in the accentuated proliferation of SMC in PAH [19]. Treatment with imatinib has been further limited by its toxicity [19]. Thus, despite numerous efforts, to date effective treatment for limiting smooth muscle hyperplasia characterizing PAH needs further development. Many of the current treatments have involved approaches such as use of calcium channel blockers, endothelin-1 receptor antagonists, tyrosine kinase inhibitors, prostacyclin analogs and phosphodiesterase-5 inhibitors [19, 21–24]. Clearly, to move toward more effective therapy, a much better understanding of the signal cascade(s) involved in the dysregulated proliferation of PAH HPASMC has to be developed such that more specific brakes on the proliferation of these cells can be achieved.

Here we report that HPASMC derived from subjects with idiopathic (i)PAH and hereditary (h)PAH are markedly hyperplastic in absence of any external growth stimulus such as growth factors or serum while they retain the SMC phenotype in primary cultures. This unstimulated proliferation occurs under non-dividing culture conditions and is promoted through MAP kinases. In presence of either PDGF-BB or FGF2 normal HPASMC also proliferate under these conditions but the proliferation is not regulated through the MAP kinase paths. This MAP kinase path promoting the dysregulated PAH SMC growth melds with the receptor tyrosine kinase signal path. Thus a combined synergistic proliferation of PAH HPASMC growth takes place in the presence of growth factors such as PDGF. Clinically, a minimally toxic regulation of the dysregulated and growth factor regulated SMC growth should result in a major advance to bringing the progress of the disease under control.

## Materials and Methods

### Reagents

The MAP kinase, mTORC1, tyrosine kinase and S6 kinase (S6K) inhibitors were purchased from Cayman Chemical (Ann Arbor, Michigan). The MTT Cell Proliferation Assay kit was purchased from ATCC (Manassas, VA). Alexa 488-conjugated anti-rabbit secondary antibody was purchased from Life Technologies (Carlsbad, CA) and Citifluor mounting medium was purchased from TED PELLA (Redding, CA). PDGF-BB (PDGF) was obtained from R&D Systems (Minneapolis, MN). The rest of the chemicals were purchased from Sigma-Aldrich (St. Louis, MO).

### Human pulmonary artery smooth muscle cell in culture

Human pulmonary artery smooth muscle cells were a generous gift of Drs. Erzurum and Comhair of the Cleveland Clinic (Cleveland, OH). The cells used in this study were obtained from non PAH (n = 3), iPAH (n = 3) and hPAH (n = 3) donor subjects [8, 25]. Subjects were identified as having PAH based on the National Institutes of Health (NIH) registry diagnostic criteria for pulmonary hypertension. Non PAH individuals had no history of pulmonary or cardiac disease or symptoms. Of the hereditary PAH SMC cells one contained a BMPR2 deletion at exons 1–8 and other had BMPR2 deletions at exons 4–5. The third contained a Smad-8 R294X mutation which is downstream of BMPR2.

Cells were isolated from elastic pulmonary arteries (>500-μm diameter) after dissection from lungs obtained at explanation during lung transplant as described by Comhair et al., 2012 [26]. Cells were cultured in complete medium containing 15 mM HEPES buffered DMEM/F12 (50:50) media (Mediatech, Manassas, VA) containing 10% fetal bovine serum (FBS) (Lonza), and 2.5% Antibiotic-Antimycotic from GIBCO (cat. no. 15240). Quiescence medium contained the same ingredients as complete medium except the FBS was reduced to 0.2%. Primary cultures of passages 6–10 were used in experiments. Approval to use these human cells was granted by the Boston University Institutional Review Board.

### Confirmation of SMC phenotype

#### a. Alpha-smooth muscle actin

Phenotype staining was performed by growing HPASMC on collagen-coated coverslips to 40% confluence. The cells were then washed 2X with 37°C PBS and fixed in 4% formaldehyde for 10 min. After incubation, cells were permeabilized with 0.4% Triton X-100 for 5 min. Following 3 washes with PBS, coverslips were incubated with 15 μg/ml goat serum in 1% BSA-PBS solution. Coverslips were incubated with anti-alpha-smooth muscle actin (SMA) antibody (Sigma-Aldrich) at (1:50) in 1% BSA-PBS solution overnight at 4°C. The next day coverslips were incubated with Alexa 488-conjugated anti-rabbit secondary antibody for 1 h at room temperature and then washed 2X with PBS. Coverslips were mounted on slides with Citifluor mounting medium and analyzed with Zeiss Fluorescence Microscope and imaging system at 400X magnification.

#### b. H-Caldesmon

To detect caldesmon levels, HPASMC were seeded in 6-well cell culture plates and grown in complete medium until reaching confluence. At that point, their medium was changed to 0.2% FBS medium overnight. The next day, medium was aspirated from each well and cells were treated with ice cold 10% TCA for 10 min. Afterwards, cells were washed 2X with ice cold PBS, lysed using 110 μl of SDS sample buffer (62.5 mM Tris-HCl (pH 6.8 at 25°C), 2% w/v SDS, 10% glycerol) and total protein harvested by cell scraping into a 1.5 ml centrifuge tube. A 10 μl aliquot of protein from each tube was quantified using a BCA assay (Thermo Fisher Scientific, Rockford, IL). Once protein was quantified, equal amounts of protein from each sample were combined with 50 mM DTT and 0.01% w/v bromophenol blue and incubated in boiling water for 5 min. Proteins were loaded and electrophoresed on SDS-PAGE with a 4% stacking and 10% separating gel. Western blots were carried out with primary rabbit polyclonal antibody for caldesmon (1:500 dilution in Blocking Buffer) and primary mouse monoclonal antibody for β-actin (Sigma-Aldrich) (1:5000 dilution in Blocking Buffer). The membranes were washed and incubated in the corresponding IRDye 700-conjugated anti-rabbit and IRDye 800-conjugated anti-mouse antibodies (LI-COR Biosciences). Membranes were visualized on an Odyssey Infrared Imaging System (LI-COR Biosciences). The low-molecular weight (L) caldesmon and high-molecular weight (H) caldesmon isoforms were visualized with the same polyclonal caldesmon antibody and identified based on their molecular weight.

### Determination of SMC proliferation

#### a. Direct cell counting procedure

HPASMC at 80–90% confluence were trypsinized and seeded onto 24 well culture plates at a density of 20,000 cells per well. Cells were allowed to attach overnight in growth medium at 37°C. The next day cell number from triplicate wells of each cell strain was determined using a Coulter Counter. The medium of the remaining wells was changed to contain 0.2% FBS. Cells were maintained in the quiescent medium containing 0.2% FBS for a designated number of days. Each day cells were detached from the wells, collected and cell number was determined. Additionally, TUNEL assays were used to detect cell apoptosis under quiescence culture conditions. Under these conditions in the presence or absence of added growth factors apoptosis proved minimal in either the PAH or non PAH cell cultures and clearly did not account for the differences in cell numbers between non PAH and PAH HPASMC.

#### b. MTT procedure

For experiments using inhibitors or growth factor stimulation, HPASMC were seeded in 96 well culture plates at a density of 3,300 cells per well. After attachment the medium was changed to 0.2% FBS medium for 4 hours and then the cells were preincubated with or without respective inhibitors for 1 h before addition of growth factors or vehicle. After 5 days, cell proliferation was measured using MTT Cell Proliferation Assay by the manufacturer’s standard procedure (ATCC, Manassas, VA). The day before MTT measurements triplicate wells on the same plate were loaded with either 0.5X, 1X, 2X or 4X the original number of cells seeded so that correlations between OD and cell number could be determined. Simultaneous experiments using cell counts and the MTT assay confirmed uniform cell proliferation results with the two assays.

### Western analysis

HPASMC were incubated overnight in 0.2% serum medium. The cells were then washed twice with ice-cold PBS. Cell lysates were prepared by addition of ice-cold RIPA buffer, 150 mM NaCl, 1.0% Igepal CA-630, 0.5% sodium deoxycholate, 0.1% SDS, 50 mM Tris, pH 8.0 (Sigma, St Louis, MO) and 1X complete protease inhibitor cocktail (Roche Applied Science, Indianapolis, IN) and centrifuged at 12,000 rpm in a microcentrifuge at 4°C for 20 minutes. The proteins were fractionated on 10% SDS-PAGE gels and western blots were carried out using antibodies against phosphorylated ERK1/2, JNK, p38MAPK and GAPDH (Cell Signaling, Danvers, MA). Proteins were detected by chemiluminescence and the film scanned with an Epson Perfection 3170 scanner using Epson Scan (version 1.22A) software.

### Statistics

Appropriate data were presented as means with standard deviations. Evaluation for statistical significance was performed with ANOVA and Turkey's *post hoc* test with p values < 0.05 considered significant.

## Results

### In vivo phenotype retention of HPASMC in culture

**Fig 1:**
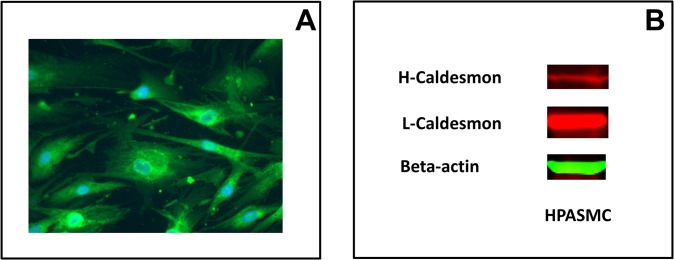
Phenotypic characterization of HPASMC in culture. (A) Immunofluorescence for alpha-SMA was merged with DAPI stain. (B) Western blot showing the presence of H and L-caldesmon in HPASMC. This figure illustrates a PAH HPASMC strain, that is representative of results obtained with various PAH and non PAH HPASMC strains.

The HPASMC were stained for alpha-SMA as a measure of homogeneous smooth muscle cell phenotype [27]. The alpha-SMA (green stain) was detected in non PAH, iPAH and hPAH cell strains examined. An example is illustrated in (Fig 1A). Since alpha-SMA is also expressed in myofibroblasts [28], the cells were also probed by western blot for H-caldesmon. The H isoform of caldesmon is only found in smooth muscle cells [29]. It is not expressed in myofibroblasts [30]. As illustrated in Fig 1B, H-caldesmon was expressed in the HPASMC.

### Cell Cycle of the HPASMC

**Fig 2:**
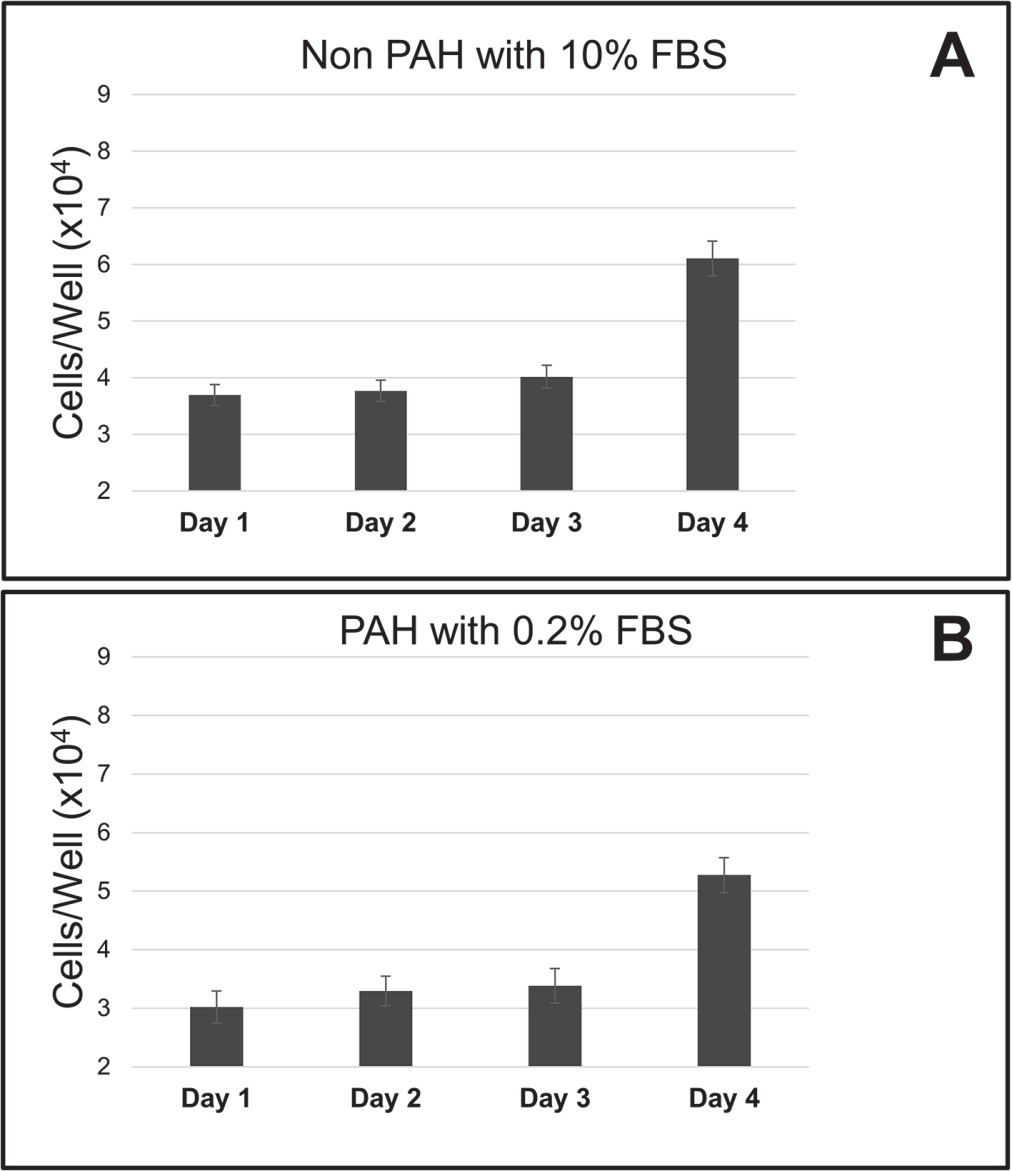
Length of HPASMC cell cycle. (A) Non PAH HPASMC were treated with the G1 arresting agent aphidicolin (10uM). After release from the block, growth was stimulated with culture medium containing 10% FBS. (B) PAH HPASMC were treated with the G1 arresting agent aphidicolin (10 uM). After release from the block, the cells were maintained in medium containing 0.2% FBS. Cell numbers were determined in triplicate each day after cell cycle blocker release. Bar graphs show the average of triplicate counts and error bars represent standard deviation.

Both the PAH and non PAH HPASMC exhibit a lengthy growth cycle of approximately 94 hours. Typical non PAH and PAH cycles are illustrated in Fig 2. When the cell cycle progression was blocked at G1/S with aphidicolin cell proliferation ceased. Following release of the block and the addition of culture medium containing 10% fetal bovine serum the synchronized non PAH cells remained at original number until day four after the block removal (Fig 2A). In the PAH cells, after aphidicolin was removed the cells continued to grow in quiescent medium. Cells doubled at approximately 94 hours (Fig 2B).

### Dysregulated effector-independent proliferation of PAH HPASMC

**Fig 3:**
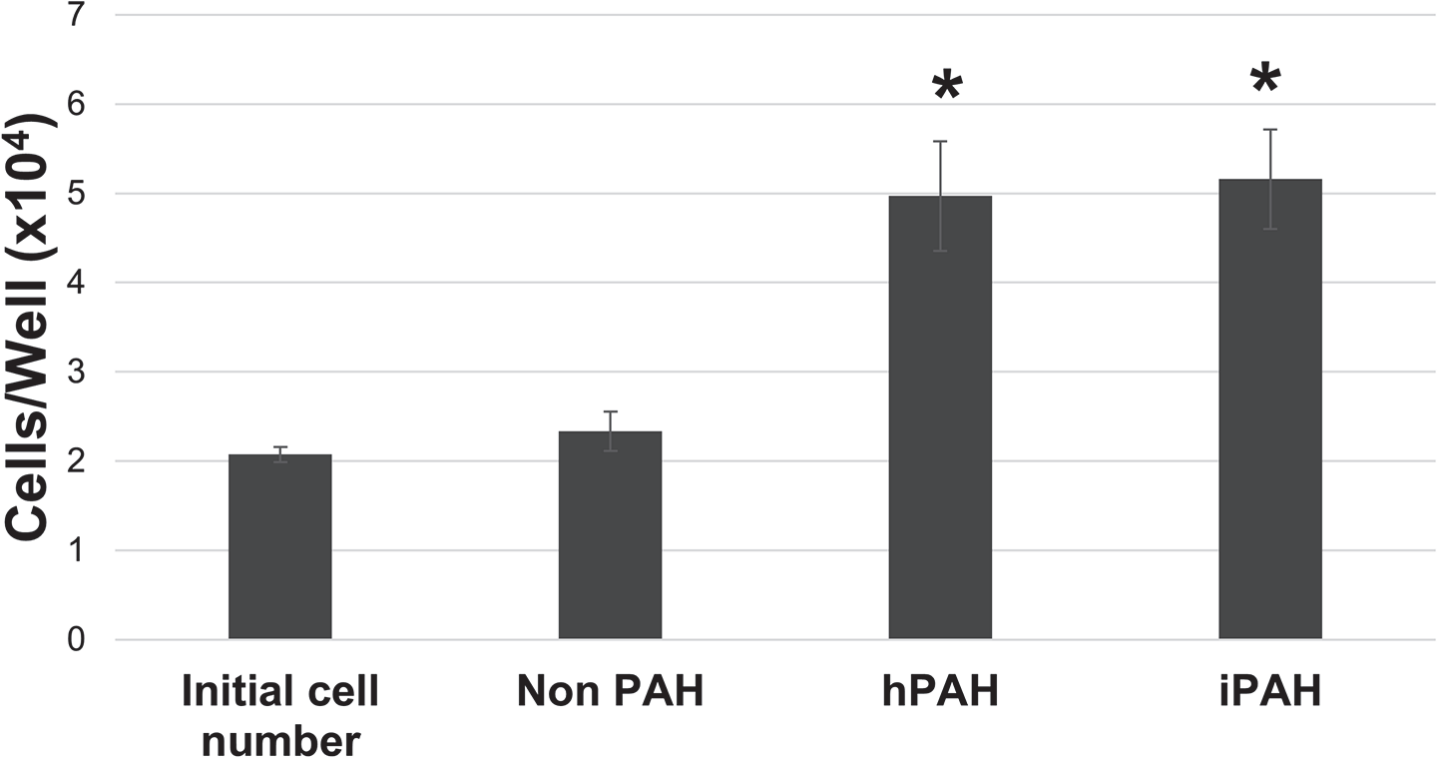
Proliferation of PAH and non PAH HPASMC in quiescence medium. Cells were cultured in quiescence medium containing 0.2% FBS. Cell number of each strain was determined in triplicate after 5 days in quiescence medium. Bar graphs represent the average cell number and the error bars are the standard deviation. *p < 0.005 vs non PAH. Results are representative of a number of experiments with various strains of non PAH, hPAH and iPAH cells.

The proliferative potentials of PAH and non PAH HPASMC were examined under quiescent, standard non growth conditions. Growth was determined at 96 h after cell plating. While the non PAH cells exhibited minimal proliferation under these conditions both hPAH and iPAH HPASMC continued to divide. This dysregulated proliferation was observed in all the PAH HPASMC samples examined. A typical experiment is illustrated in Fig 3. As illustrated, over the duration of the experiment the non PAH cells showed an insignificant increase in number. The PAH cells obtained from both iPAH and hPAH lungs more than doubled in number. Conditioned medium from either non PAH or PAH cell cultures equally caused a moderate increase in non PAH cell numbers over 96 h, but well below the level of the PAH cell growth (data not shown).

### MAP kinase involvement in the dysregulated HPASMC proliferation

**Fig 4:**
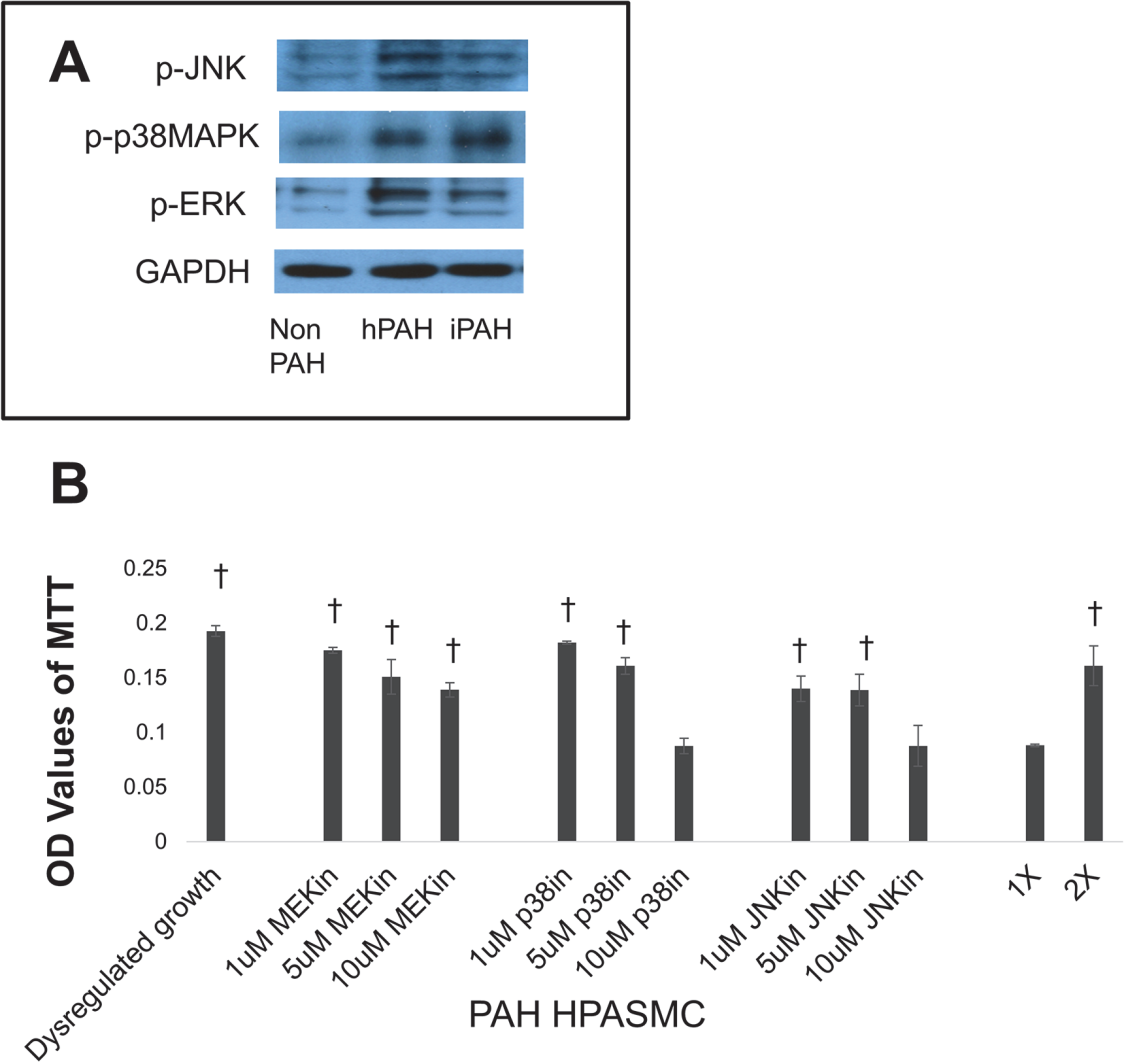
PAH HPASMC proliferation via MAP kinases. (A) Western blot showing levels of phospho-JNK, phospho-p38MAPK, phospho-ERK and GAPDH after overnight incubation of the cells in medium containing 0.2% FBS. (B) Twenty four hours after seeding, the growth medium was replaced with quiescence medium and inhibitors (MEKin = U0126; p38in = SB203580; JNKin = SP600125) at respective concentrations were added to the culture. Cell proliferation was determined 5 days later using the MTT assay. Bar graphs represent the average OD values from triplicate wells. The error bars represent standard deviation. This figure shows results from an hPAH cell strain and is representative of results obtained from other PAH cell strains. †p < 0.001 vs 1X cell number.

Previous studies have shown that MAP kinases play a pivotal role in excessive lung cell proliferation in certain cancers [31]. To address the possible involvement of MAP kinases in the observed dysregulated HPASMC proliferation, we initially compared basal activated expression under quiescent growth conditions in non PAH, hPAH and iPAH cells for activated ERK, p38 MAPK and JNK. The results indicated that the levels of activated ERK, JNK and p38 MAPK were markedly higher in both iPAH and hPAH cells compared to non PAHs (**Fig 4A**). Next, participation of MAP kinases in the dysregulated proliferation was examined. Phosphorylation of MEK/ERK, p38 MAPK and JNK was blocked. A typical result is illustrated in (Fig 4B). The dysregulated growth approximately doubled compared to the original cell number shown as 1X and the original plated doubled number (2X). Inhibiting either the formation of phosphorylated p38 MAPK or JNK prevented the increase in the dysregulated growth with the cells remaining at the 1X level. On the other hand, the influence of ERK on the proliferation appears only marginal.

### Signal cascade driving growth factor promoted proliferation

**Fig 5:**
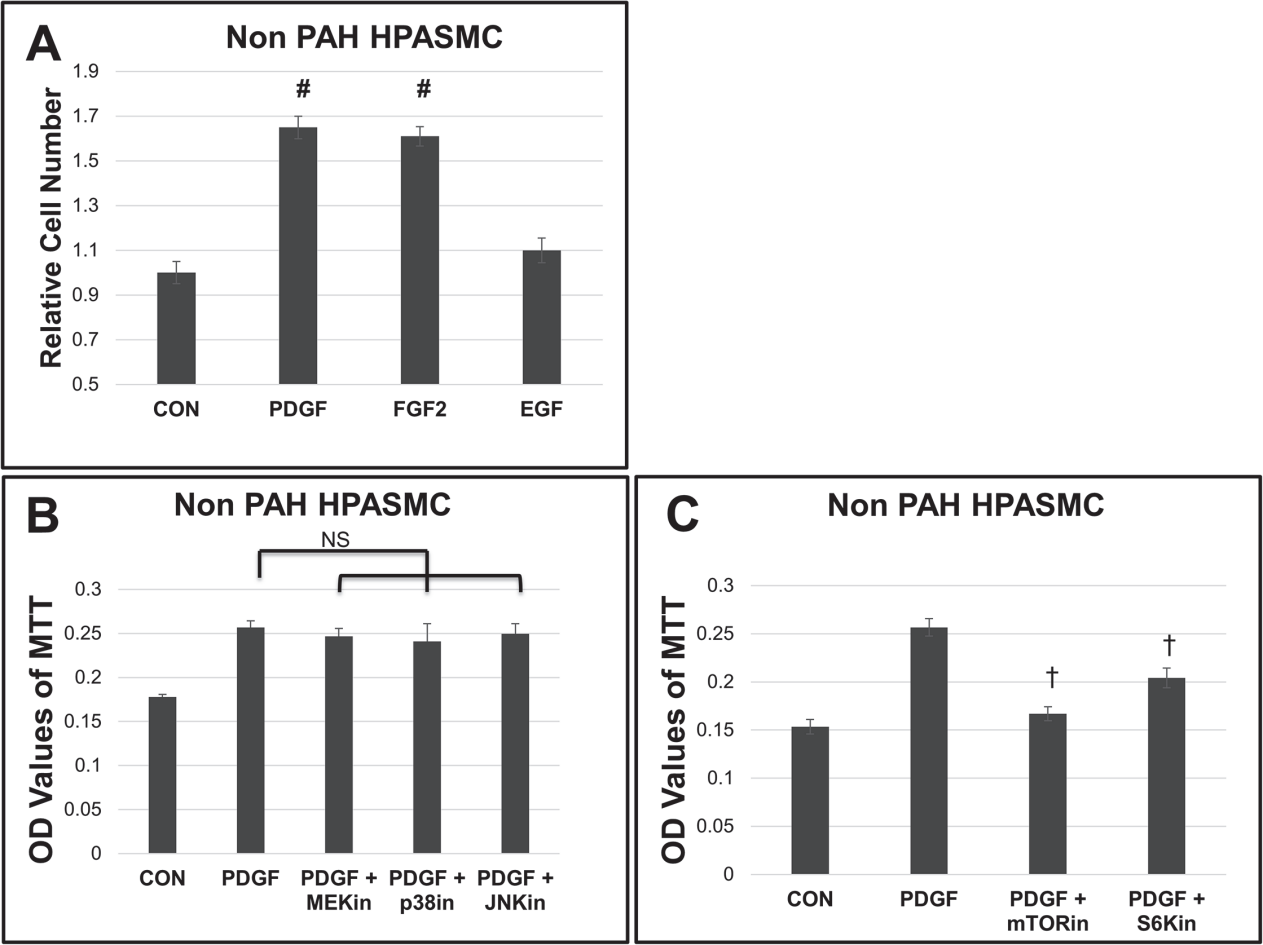
Growth factor proliferative signaling in non PAH HPASMC. (A) Non PAH HPASMC were cultured in medium containing 0.2% FBS in the presence of 10 ng/ml PDGF, FGF2, or EGF over 5 days. Cell number was determined with a Coulter Counter. Bar graphs represent the mean from triplicate wells and the error bars are standard deviation. (B) After attachment over 24 hours the growth medium was replaced with quiescence medium and the cells preincubated for 30 min with 10 uM of either U0126 (MEKin), SB203580 (p38in), or SP600125 (JNKin) (A) or 10 ng/ml rapamycin (mTORin) or 10 uM PF4708671 (p70S6Kin) (C) as indicated cells were then treated with 10 ng/ml PDGF, FGF or EGF. Cell proliferation was determined 5 days later by MTT assay. Bar graphs represent the average OD values of triplicate wells. The error bars represent standard deviation. #p < 0.01 vs nonPAH; NS, non-significant; †p < 0.001 vs PDGF. Shown is a representative result from a non PAH strain.

PDGF, FGF2, and EGF are known promoters of cell proliferation of SMC. Their signaling cascades were examined in non PAH HPASMC. Results are illustrated in Fig 5. PDGF and FGF2 promoted the largest increase of SMC growth in culture, while EGF promoted a more modest increase in proliferation across cell strains tested (**Fig 5A**). Since PDGF promoted a more consistent growth increase, we continued our experiments looking at common signaling cascades between PDGF and the dysregulated growth. In Fig 5B, none of the MAP kinase (MEK/ERK, JNK or p38 MAPK) inhibitors were able to abrogate PDGF stimulated proliferation in the non PAH HPASMC. Instead, inhibition of mTOR or S6K activation significantly reduced PDGF promoted cell proliferation (Fig 5C).

### Non Involvement of growth factor receptors in the dysregulated HPASMC

**Fig 6:**
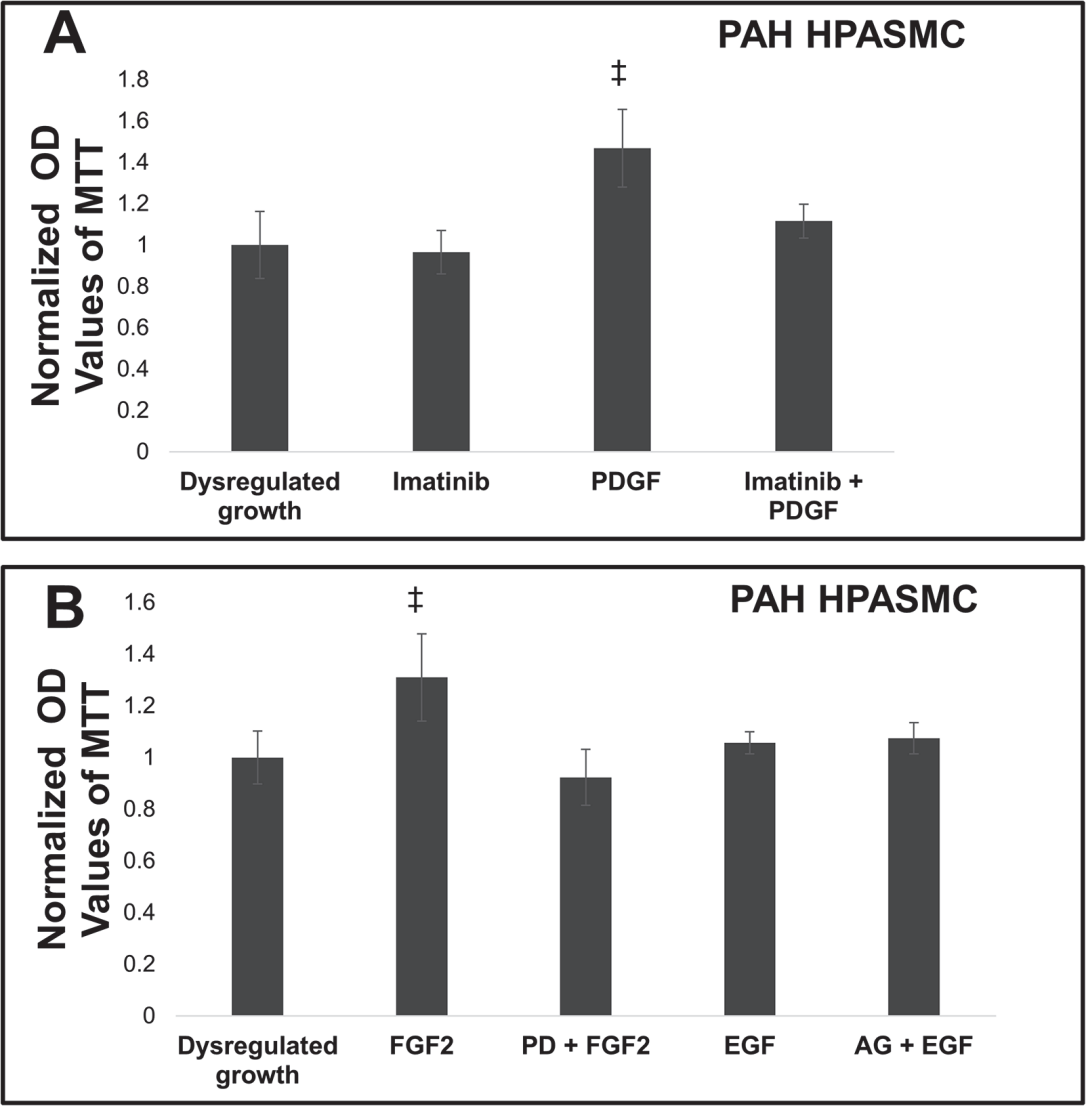
Effect of receptor tyrosine kinase activity inhibition on growth of PAH HPASMC. Twenty four hours after seeding the growth cell medium was replaced with quiescence medium. Cells were preincubated for 30 min with 5uM imatinib and then treated with or without 10 ng/ml PDGF, FGF or EGF (A) preincubated for 30 min with 100 nM PD 173074 and then treated with or without 10 ng/ml FGF2 or preincubated for 30 min with 100 nM AG-1478 and then treated with or without 10 ng/ml EGF (B). Cell proliferation was determined 5 days later using the MTT assay. Bar graphs represent the average OD values from triplicate wells. The error bars are standard deviation. This figure represents a result from an iPAH strain and is representative of results obtained from other PAH cell strains. ‡p < 0.05 vs dysregulated growth.

The PAH HPASMC do not require mitogenic stimulation to proliferate. However, when PDGF was added, it did augment their dysregulated growth by approximately 1.5 fold. Results are shown in Fig 6. To illustrate whether this augmentation takes place through the PDGF receptor an inhibitor of the activated PDGF receptor phosphorylation, imatinib, was also used in the presence and absence of PDGF. Imatinib had no effect on the dysregulated growth, but basically abrogated the PDGF augmented growth. The example shown in Fig 6A was from an iPAH HPASMC culture. When investigating the effects of FGF2 and EGF on dysregulated growth, FGF2 did augment growth while EGF did not. Moreover, neither inhibitors against FGF2 (PD 173074) or EGF (AG-1478) receptors had any effect on the dysregulated growth, but FGF receptor inhibition did block FGF2 stimulated growth (**Fig 6B**).

### Involvement of mTOR/S6K as downstream target of the MAP kinases

**Fig 7:**
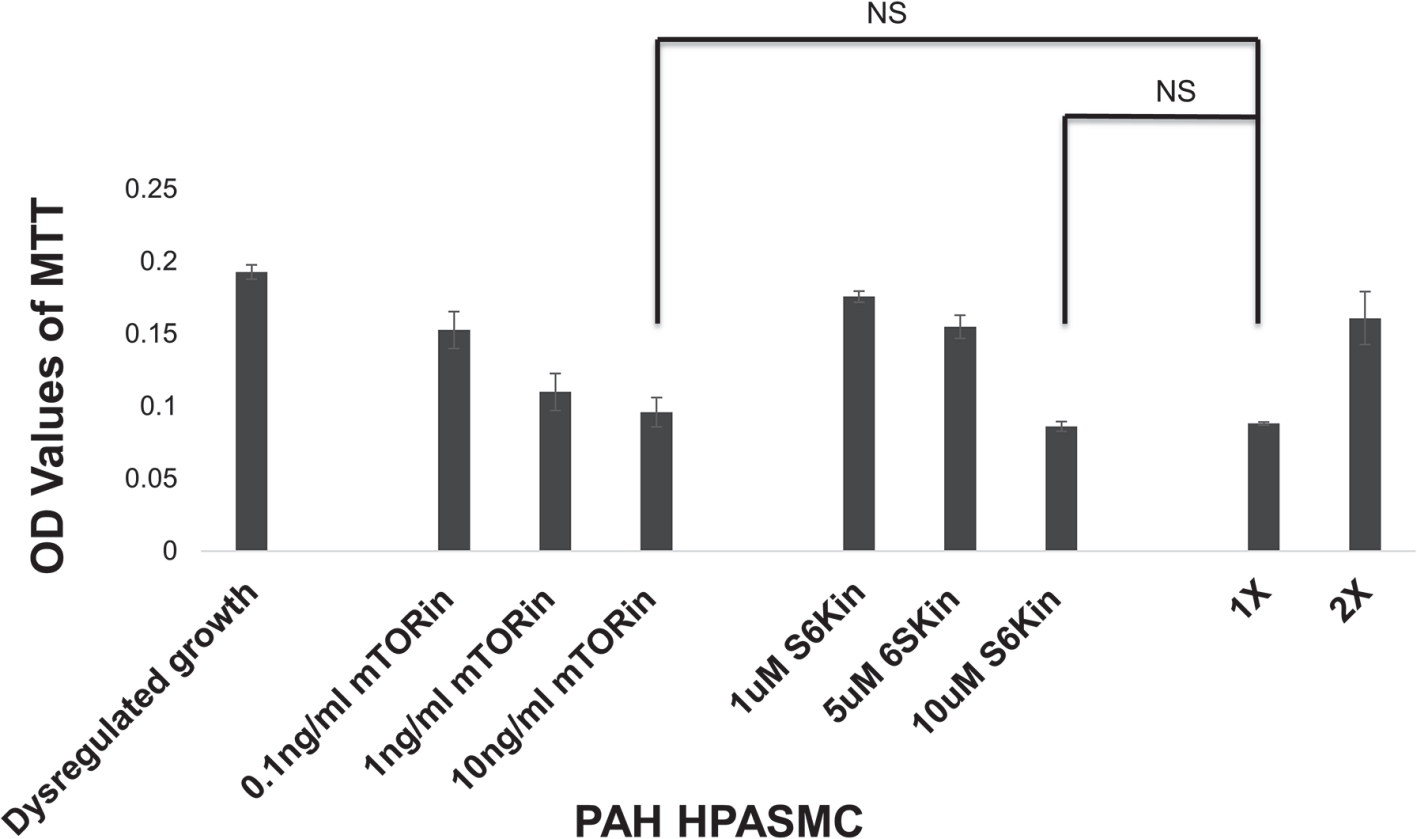
Regulation of PAH HPASMC proliferation at mTOR. Twenty four hours after seeding the growth medium was replaced with quiescence medium. Inhibitors (mTORin = rapamycin; p70S6Kin = PFPF4708671) at respective concentrations were added. Cell proliferation was determined 5 days later using the MTT assay. Bar graphs represent the average OD values from triplicate wells. The error bars are standard deviation. This figure is from an hPAH cell strain and is representative of results obtained from other PAH cell strains. NS = non-significant.

**Fig 8:**
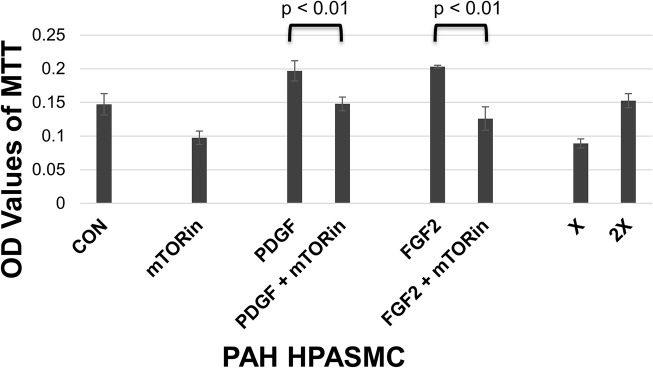
PAH HPASMC dysregulated and growth factor stimulated proliferation. Twenty four hours after seeding, medium was replaced with quiescence medium. Cells were preincubated for 30 min with 10 ng/ml rapamycin (mTORin) and then treated with or without 10 ng/ml PDGF or FGF2. Cell proliferation was determined 5 days later using the MTT assay. Bar graphs represent the average OD values from triplicate wells. The error bars are the standard deviation. This figure is from an hPAH cell strain and is representative of results obtained from other PAH cell strains.

Since mTOR and S6K inhibitors were effective at abolishing PDGF stimulated proliferation in non PAH HPASMC, we tested their effects on the dysregulated growth of PAH HPASMC. We used inhibitors against mTOR (rapamycin) and S6K (PF4708671) at three different concentrations to determine their involvement in HPASMC dysregulated proliferation. Both inhibitors greatly restricted the dysregulated proliferation of PAH HPASMC. A representative example from an hPAH sample is shown in Fig 7. Since mTOR inhibition blocked PDGF promoted proliferation in non PAH HPASMC and dysregulated proliferation in PAH HPASMC, we also wanted to see its effect on growth factor stimulated HPASMC. **Fig 8** shows that mTOR inhibition reduces both PDGF and FGF2 stimulated proliferation in PAH HPASMC.

## Discussion

PAH is associated with an activated proliferation of cells within the pulmonary artery [1]. The smooth muscle cell proliferation and accompanied migration thickens the vascular media, invades the intima and participates in the formation of plexiforms [32]. These events contribute to an elevated pulmonary vascular pressure. Smooth muscle cells from human pulmonary arteries in primary cultures are now being utilized as a model for the smooth muscle behavior in PAH. In culture these cells retain much of their *in vivo* characteristics such as expression of alpha-SMA and H-caldesmon. They also retain their contractile characteristics as shown by constriction in response to ET-1 [8, 33]. As illustrated here the PAH SMC undergo a sizable autologous growth from both hPAH and iPAH. This growth takes place under conditions where non PAH cells do not divide reflecting the in vivo loss of homeostasis and resulting hyperplasia in PAH. Moreover, in culture the dysregulated growth is not due to the production of cellular secretions into the culture medium. Conditioned medium from PAH HPASMC did not stimulate growth of non PAH HPASMC more than conditioned medium from non PAH HPASMC. This suggests that endogenous events are taking place within the SMC of PAH patients.

Recent efforts to better understand the regulation of this segment of remodeling have focused on tyrosine kinase receptors [14–17]. One such effort is exemplified by clinical trials of imatinib, a modulator of the catalytic sites of the PDGF receptor, as a modulator of PAH [18, 19]. Our results here show that imatinib indeed abrogates the PDGF accentuated proliferation, but has no effect on the dysregulated growth. This may offer a clue as to the limitations that imatinib has shown in clinical trials [19]. However, given that PDGF levels are upregulated in pulmonary arteries of patients with iPAH [34], it may still be important to mute the PDGF growth effect in combination with other drug targets for PAH. However, caution must be taken not to entirely block the actions of PDGF on other critical physiologic functions. Therefore, muting of specific PDGFR signal cascades regulating SMC growth in PAH would be advantageous. This may be possible through approaches such as cell penetrating peptides (CPP). Initial efforts to selectively modulate PDGF receptor signaling have proved successful [35].

While the PDGF path to HPASMC growth has been shown to be taking place via PI3K/mTOR [36–38], we are finding that the dysregulated cell growth is promoted principally through p38 MAPK and JNK. Furthermore, we are finding that the PAH cells continue to respond to PDGF with additional synergistic proliferation adding to the dysregulated growth. However, the growth response to PDGF in the PAH cells is no greater than taking place in the normal cells. Our results further indicate that FGF2 but not EGF can also participate in this synergistic overgrowth of the HPASMC via the mTOR route.

Here we show for the first time that the observed PAH dysregulated SMC growth in HPASMC is cascading via the MAP kinase paths. At this time it is not clear what is activating the p38 MAPK and JNK to promote the dysregulated growth. MAP kinases have been shown to be involved in the growth response to a number of PASMC growth effectors [5–7]. Constitutively active forms of p38 MAPK and JNK have been reported to promote uncontrolled proliferation in other cells types [39, 40]. Increased MAP kinase activation has been associated with remodeling of pulmonary arteries in both hypoxia and monocrotaline rat models of pulmonary hypertension [41–43]. Another possibility is that overexpression or constitutive activity of a kinase upstream of and simultaneously activating JNK and p38 MAPK is taking place in the PAH cells. For example, simultaneous activation of JNK and p38 MAPK by the MLK family of kinases has been reported [44, 45]. A recent study by Brown and colleagues demonstrated that transgenic mice with global knockout of MAP kinase kinase kinase-2 (MEKK2) had reduced right ventricle hypertrophy compared to wild-type controls under chronic hypoxia [46]. Therefore, MEKK2 or a similar kinase upstream of MAP kinases could be activing JNK and p38 MAPK in HPASMC in PAH. Alternatively, PAH SMC possess an increased intracellular Ca^2+^ uptake [5,6]. This increased Ca^2+^ translocation may be activating the JNK/p38 MAPK paths. Another possible activator is the glycolytic path which was shown to be utilized in the PAH cells as an energy source [36].

Our results suggest that the MAP kinase paths join the PDGF stimulated growth path at mTOR/S6K. Participation of mTOR in the dysregulated growth has been reported very recently by Goncharov and co-workers (2014) [36]. In fact, a very recent report suggests that the mTOR activation represses the expression of FoxO3a, a transcription factor known to activate genes for growth arrest [47]. Another recent study implicates downregulation of FoxO1 expression in pulmonary vessels and PASMC from human and experimental models of PAH as being at least partly responsible for the increased proliferation and decreased apoptosis of PAH PASMC [48]. The downregulation of FoxO1 has been shown to be induced by PDGF cascading through PI3K/Akt [48]. It remains to be seen whether JNK and/or p38 MAPK are also directly involved in the regulation of any of the activities of these transcription factors in PAH. Interestingly, using DNA microarray we found sizable increases in mRNA expression of factors associated with the promotion of SMC growth and cell cycle progression in PAH cells from both h and iPAH subjects [49]. These differences in gene expression patterns between PAH and non PAH HPASMC indicated upregulation of another Forkhead transcription factor, FOXM1. FOXM1 has been shown to be upregulated in multiple cancer types causing increased proliferation, survival and migration [50]. FoxM1 is also negatively regulated by FoxO transcription factors [51, 52]. These factors may prove to be viable candidates for modulation of their action in PAH.

**Fig 9:**
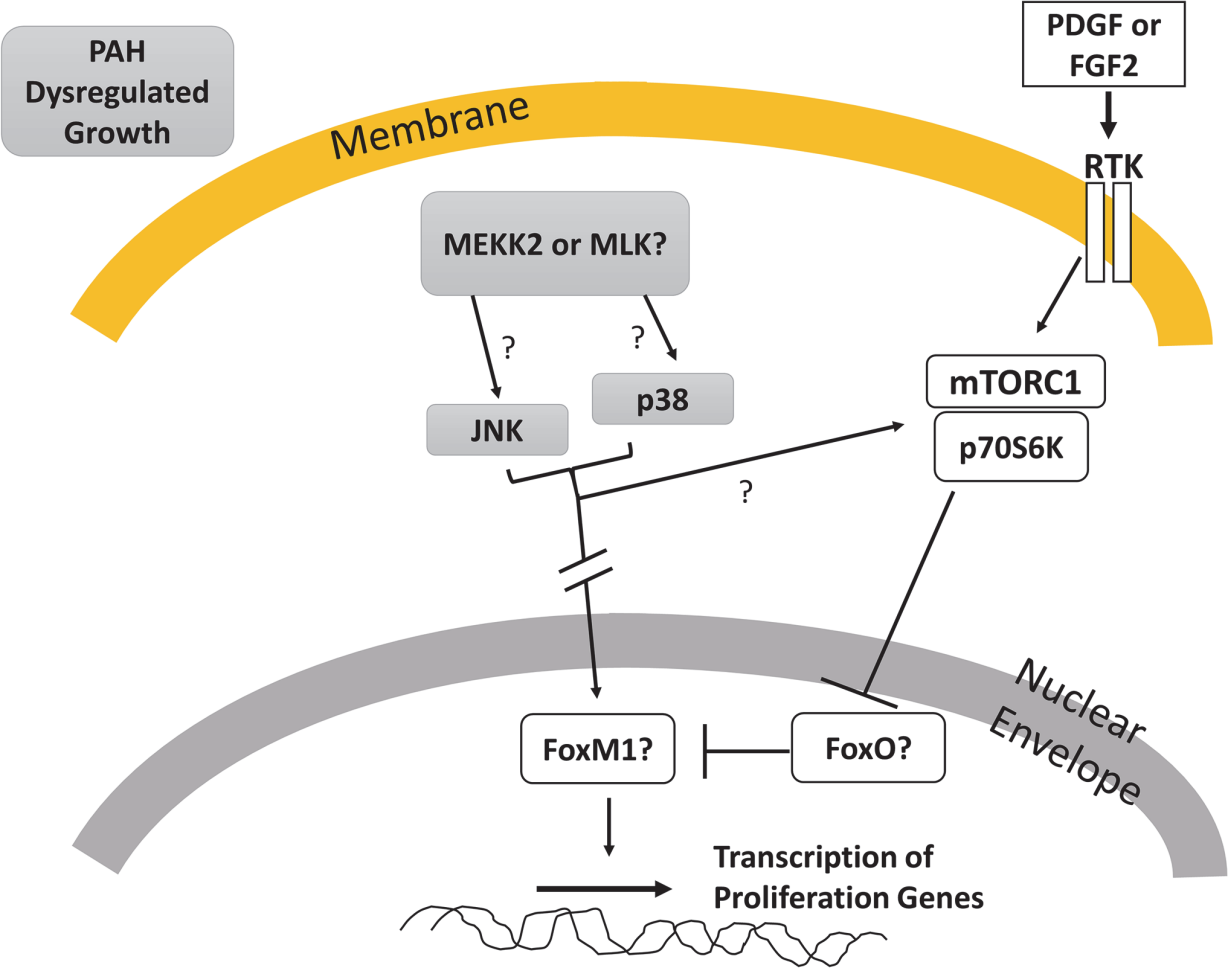
HPASMC signaling cascades for PAH dysregulated cell proliferation.

A projected scheme involving the dysregulated MAP kinase cascade is sketched in **Fig 9**. In PAH HPASMC JNK and p38 MAPK may be phosphorylated by MEKK2 or MLK (or other MAPK superfamily members) further channeling into transcription factors in the nucleus such as, FOXM1. Alternatively, JNK and p38 MAPK may cascade directly into mTOR/S6K which then inhibits FOXO activity so FOXO cannot negatively regulate FOXM1. This mTOR/S6K path is also involved in growth factor promoted proliferation.

Results in sum illustrate that control of proliferation in PAH HPASMC encompasses both receptor tyrosine kinase stimulated and dysregulated facets. Initiation of the dysregulated growth utilizes both JNK and p38 cascades. Since inhibiting either of these MAP kinases or mTOR activation abolishes the dysregulated growth and inhibiting the mTOR activation also abolishes the tyrosine receptor kinase stimulated growth, at this time it appears that the activated MAP kinases cascade through mTOR and then S6K. However, it is possible that parallel pathways utilizing both mTOR and MAP kinases are required to obtain the dysregulated growth. These points need further clarification. Also, clearly, the sample number in this communication is small. Therefore at this time any suggestion that our findings will lead to a therapeutic approach is premature and awaits much more in depth investigation.
